# Digital education for cardiac care readiness among coronary heart disease patients in low- and middle-income countries: a scoping review

**DOI:** 10.21542/gcsp.2026.27

**Published:** 2026-06-30

**Authors:** Nuridah, Rosyidah Arafat, Andi Masyitha Irwan

**Affiliations:** Hasanuddin University, Makassar, South Sulawesi, Indonesia

## Abstract

**Introduction:** Coronary heart disease (CHD) is the leading cause of cardiovascular mortality worldwide, with the greatest burden concentrated in low- and middle-income countries (LMICs). Patient readiness—encompassing knowledge, self-efficacy, and self-care behaviour—is a critical determinant of post-discharge outcomes. Digital education interventions offer an innovative, resource-efficient approach to improving CHD patient readiness in LMICs; however, the available evidence has not been systematically mapped.

**Methods:** This scoping review followed the Arksey and O’Malley framework and was reported in accordance with the PRISMA Extension for Scoping Reviews (PRISMA-ScR). A systematic search was conducted across four databases—PubMed, ScienceDirect, Wiley Online Library, and the Cochrane Library—for publications from 2019 to 2026. The most recent searches were run on 1 March 2026 and supplemented by manual screening of reference lists.

**Results:** Eleven studies were included, comprising 7 full RCTs (63.6%), 1 pilot RCT (9.1%), 2 RCT protocols (18.2%), and 1 feasibility study (9.1%). Geographic distribution included China (*n* = 4; 36.4%), Iran (*n* = 4; 36.4%), Colombia (*n* = 1; 9.1%), Tunisia (*n* = 1; 9.1%), and Brazil (*n* = 1; 9.1%). The most frequently used primary delivery platforms were mobile applications/smartphones (*n* = 3; 27.3%), WeChat (*n* = 2; 18.2%), WhatsApp (*n* = 2; 18.2%), Web-based/online platforms (*n* = 2; 18.2%), augmented reality (*n* = 1; 9.1%), and digital multimedia (*n* = 1; 9.1%). The most commonly measured outcomes were self-efficacy (*n* = 4; 36.4%), quality of life (*n* = 3; 27.3%), disease knowledge/illness perception (*n* = 3; 27.3%), medication adherence/self-management (*n* = 3; 27.3%), and clinical events (*n* = 1; 9.1%). Discharge readiness was not measured as a primary outcome in any included study.

**Conclusion:** Digital education interventions show promising potential for improving the components of CHD patient readiness in LMICs; however, the evidence remains concentrated in China and Iran. Research specifically targeting discharge readiness with validated instruments is urgently needed across diverse LMIC settings, particularly in Southeast Asia, sub-Saharan Africa, and Latin America.

## Introduction

Cardiovascular diseases (CVDs) remain the leading cause of mortality and disability worldwide. The number of cases nearly doubled between 1990 and 2019, from 271 million to 523 million, while annual deaths rose to 18.6 million^1^. Coronary heart disease (CHD) is the dominant component of this burden. More than 80% of cardiovascular deaths occur in low- and middle-income countries (LMICs), driven by rapid urbanization, population ageing, and a high prevalence of modifiable risk factors^1,2^. LMICs also face compounded challenges from inadequate health service infrastructure, workforce shortages, and limited access to specialized cardiovascular care^5,6^.

Following an acute cardiac event or revascularization procedure, patient readiness—encompassing disease knowledge, self-efficacy, and self-care behaviour—is critical for optimal post-discharge outcomes^3,8^. Suboptimal readiness is strongly associated with non-adherence to secondary prevention therapies, poor quality of life, increased risk of recurrent acute coronary events, and preventable hospital readmissions^3,8,26^. Despite the proven benefits of comprehensive cardiac rehabilitation (CR) and secondary prevention programmes, their uptake remains very low: fewer than 20% of eligible patients participate worldwide, and even fewer in resource-constrained LMIC settings^4,9^.

To bridge this gap, digital health interventions—such as mobile applications, social media platforms (e.g., WeChat, WhatsApp), and web-based telerehabilitation—have emerged as innovative, resource-efficient models for delivering health education^4,7,9^. The mapped evidence suggests that digital education interventions have the potential to support CHD-related knowledge, cardiac self-efficacy, medication adherence, and self-care behaviour across diverse patient populations. For example, a nurse-led eHealth cardiac rehabilitation programme in China was associated with improvements in cardiac self-efficacy, health behaviours, and quality of life among post-discharge CHD patients^8,10^. Similarly, a WhatsApp-based education programme in Brazil showed high feasibility for supporting exercise self-efficacy and sleep quality among cardiac patients in a low-resource setting^11^.

However, despite the growing body of primary studies evaluating digital health in cardiovascular care, there has been no comprehensive evidence mapping focused specifically on digital education interventions targeting CHD patient readiness in LMICs. This review therefore aims to systematically map the available evidence and inform future research priorities, clinical practice, and digital health policy in these settings.

## Methods

### Study design and framework

This scoping review followed the methodological framework proposed by Arksey and O’Malley^12^ and was reported in accordance with the PRISMA Extension for Scoping Reviews (PRISMA-ScR)^13^. A protocol was developed a priori to prespecify the objectives, eligibility criteria, and analytic approach, reducing the risk of selective reporting. A scoping review methodology was chosen because the aim was to map the breadth and nature of the available evidence rather than to assess methodological quality or synthesize effect sizes.

### Search strategy and information sources

A systematic search was conducted across four major electronic databases: PubMed (MEDLINE), ScienceDirect (Elsevier), Wiley Online Library, and the Cochrane Library (CENTRAL). The search was limited to publications from January 2019 to December 2025 to keep the findings relevant to current digital technologies and the post-COVID-19 health context^25^. All database searches were last run on 1 March 2026. No language restrictions were applied at the initial search stage; however, studies were included only if the full text was available in English or Indonesian.

### Study selection process and data extraction

All records identified from databases were imported into Rayyan QCRI for deduplication and collaborative screening. To ensure compliance with the PRISMA-ScR framework^13^, all records were screened and strictly evaluated against the exact same inclusion and exclusion criteria.

Inclusion criteria comprised: (1) adult patients ≥18 years with a diagnosis of CHD; (2) any digital education component; (3) at least one measurable patient readiness outcome; (4) study conducted in LMICs according to the World Bank classification current at the time of this review (2024–2025 period)^14^; and (5) study design of RCT, quasi-experimental, pilot study, feasibility study, or published clinical trial protocol. Studies were excluded if they involved patients with non-CHD cardiovascular conditions only, were conducted exclusively in high-income countries, lacked a digital education component, or were reviews, editorials, or conference abstracts without full-text data. To ensure consistent methodological mapping, the 11 included studies were classified using a standardized study design typology consisting of three categories: (1) Randomized Controlled Trials (RCTs), defined as prospective studies with random group allocation, encompassing full RCTs, pilot RCTs, and RCT protocols ; (2) Quasi-experimental studies, defined as non-randomized interventional studies; and (3) Feasibility studies, defined as preliminary descriptive evaluations of an intervention’s viability . No quasi-experimental studies were ultimately included in the final map.

### Ethical considerations

As a scoping review of previously published, publicly available literature, this study did not involve direct interaction with human or animal subjects or access to confidential patient records. Formal ethics committee review and approval were therefore not required. We confirmed that all primary studies included in this review reported obtaining the appropriate institutional ethical approvals and informed consent prior to publication.

## Results

### Study selection process

**Figure 1.**
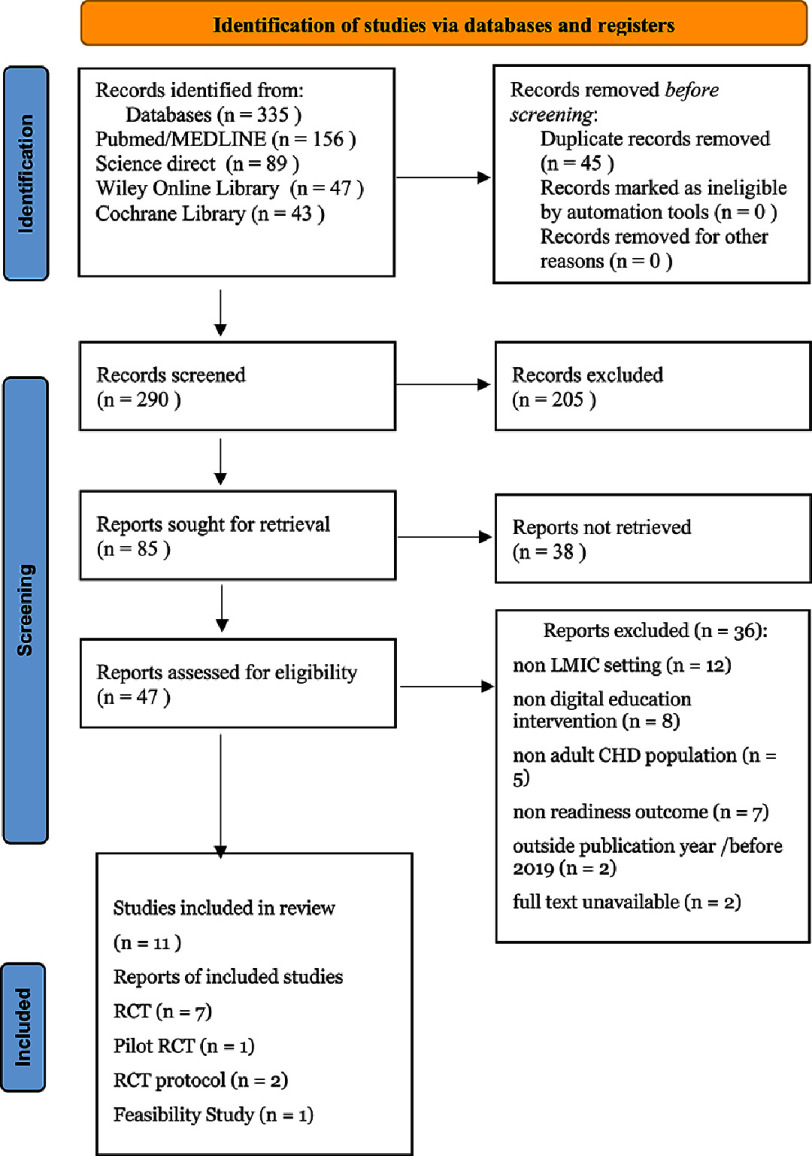


The systematic search across four electronic databases, run on 1 March 2026 for publications from January 2019 to December 2025, identified 335 records. Full-text review was conducted on 47 articles: 44 retrieved from the databases and 3 identified through manual searches and expert consultation. After application of the inclusion and exclusion criteria, 11 studies were included in the review. The study selection process is documented in the PRISMA 2020 flow diagram^15^ (Figure 1).

### Characteristics of included studies

Based on the standardized typology, the 11 included studies fell into three design categories: 9 were classified as randomized controlled trials (7 full RCTs, 1 pilot RCT, and 1 RCT protocol; 81.8%), 1 was a feasibility study (9.1%), and 1 was a published RCT protocol (9.1%).

Geographically, the studies were concentrated in upper-middle- and lower-middle-income countries. China (*n* = 4, 36.4%) and Iran (*n* = 4, 36.4%) were the most represented, followed by Colombia, Tunisia, and Brazil (*n* = 1 each, 9.1%). Under the World Bank classification current at the time of the review (2024–2025), all included studies originated from middle-income countries (upper-middle or lower-middle), and applying this classification did not alter the eligibility of any study—see Table 1.

**Table 1:** Table of master characteristics table of included studies (*n* = 11).

No.	Author (Year) [Ref]	Country	Study design	Population	Primary digital platform	Readiness outcome/key findings
1	Li, Y., et al. (2022)^16^	China	Pilot RCT	CHD patients	Mobile app (DTx)	Adherence and clinical outcomes improved
2	Jacome-Hortua, A. M., et al. (2024)^17^	Colombia	RCT Protocol	Cardiac rehabilitation patients	WhatsApp	Knowledge, physical activity, quality of life (SF-36)
3	Ahmadi, Z., et al. (2022)^18^	Iran	RCT	ACS patients	Digital multimedia	Self-efficacy ↑18.7 points (*p* < 0.001); self-esteem ↑
4	Ghali, H., et al. (2024)^19^	Tunisia	RCT Protocol	CHD patients post-discharge	Web-based digital platform	Prevention of major cardiovascular events (MACE)
5	Salavati, K., et al. (2025)^20^	Iran	RCT	Post-PCI patients	Mobile app	Illness perception ↑12.4 points (*p* = 0.002); self-care ↑
6	Kang, G., et al. (2023)^21^	China	RCT	Stable CAD patients	WeChat	Knowledge ↑8.3 points (*p* < 0.001); quality of life ↑11.5 points
7	Su, J. J., & Yu, D. S. (2021)^10^	China	RCT	Hospitalized CHD patients	WeChat	Self-efficacy ↑14.6 points (*p* < 0.01); quality of life ↑9.8 points
8	Nikraftar, F., et al. (2022)^22^	Iran	RCT	CAD patients	Smartphone app	Acceptability, feasibility, and effectiveness improved
9	dos Santos, R. Z., et al. (2023)^11^	Brazil	Feasibility study	Cardiac patients in CR program	WhatsApp	Exercise self-efficacy ↑2.8 points; sleep quality ↑; depression ↓
10	Moghaddam, N. G., et al. (2023)^23^	Iran	RCT	Post-CABG patients	Augmented reality (AR)	Cardiovascular management self-efficacy ↑ significantly (*p* < 0.001)
11	Ma, L.-C., et al. (2024)^24^	China	RCT	Stage III CR patients	Web-based online platform	Self-management behavior ↑ (*p* < 0.05); anxiety & depression ↓

Because most of the included trials were small to moderate in size and reported sample sizes inconsistently, an aggregate sample size was not calculated for this scoping review. Instead, the mapped evidence is interpreted primarily in terms of study design, setting, and outcome domains, reflecting the exploratory nature of scoping reviews and the early-stage development of digital education interventions for CHD readiness in LMICs.

### Digital education platforms used

The digital education platforms used in the 11 included studies were heterogeneous, and many interventions combined more than one modality. Mobile applications or smartphone-based tools (for Android/iOS or generic smartphones) were the most frequently described, appearing in three studies from China and Iran^6,20,22^. WeChat-based interventions were reported in two Chinese studies, one nurse-led eHealth cardiac rehabilitation programme and one health-education trial in stable coronary artery disease^10,21^. WhatsApp-assisted education was used in interventions from Colombia and a low-resource Brazilian setting, where it was highlighted as a feasible, acceptable option for patient engagement^11,17^.

Web-based or online platforms, including a nurse-managed eHealth portal and an online learning environment for stage III cardiac rehabilitation, were each evaluated once^10,24^. Digital multimedia education and an augmented-reality (AR)–based phase I cardiac rehabilitation programme provided more immersive, interactive learning experiences in two Iranian trials^18,23^. Finally, one study in Tunisia evaluated an integrated digital monitoring and education platform for coronary heart disease^19^.

### Patient readiness outcomes measured

Self-efficacy was the most frequently measured outcome in this review, assessed in 4 studies (36.4%). Quality of life (QoL) and disease knowledge (or illness perception) were each measured in 3 studies (27.3%). Medication adherence and self-management behavior were assessed in 3 studies (27.3%). Most strikingly, none of the 11 studies explicitly measured discharge readiness as a primary outcome using validated instruments such as the Readiness for Hospital Discharge Scale (RHDS).

## Discussion

### Mapped utility and reported outcomes of digital platforms

This scoping review maps a range of digital education interventions, with mobile applications, WeChat and WhatsApp the most frequently used modalities in LMICs. Across the included studies, authors generally reported positive trends in the utility of these interventions.

Purpose-built mobile applications, such as self-management digital therapeutics (DTx), were described as potential tools for supporting long-term adherence to guideline-recommended medications through automated tracking, personalized reminders, and lifestyle-intervention plans^16^. Social media platforms—particularly WeChat^10,21^ in China and WhatsApp^11,17^ in Latin America—were frequently highlighted as feasible, accessible options that may reduce barriers to technology adoption for patients.

For example, a nurse-led eHealth cardiac rehabilitation programme using WeChat was reported to facilitate goal-setting, peer support, and real-time professional feedback, with the authors observing improvements in health behaviours, self-efficacy, and health-related quality of life^10^. Similarly, a feasibility study involving cardiac patients in a low-resource setting suggested that WhatsApp interventions could achieve high patient satisfaction and engagement^11^, though larger controlled trials are needed before these findings can be generalized.

Augmented reality (AR) also emerged in the mapped literature as a novel approach to phase I cardiac rehabilitation, providing immersive, interactive physical training; one included trial reported that AR use was associated with improved cardiovascular-management self-efficacy among post-CABG patients^23^. For older populations, or those in regions with limited internet infrastructure, simpler technologies such as SMS remain widely available and affordable, and studies evaluating SMS reported positive associations with reduced composite clinical events and improved self-care behaviour^5,7^. Consistent with the nature of a scoping review, however, these reported outcomes indicate promising potential rather than definitive evidence of clinical effectiveness.

### Facilitators and barriers in LMIC contexts

The implementation of digital health technologies in LMICs faces distinct challenges and drivers compared with high-income countries, owing to differences in resources, health care infrastructure, and the digital divide^6,25^. Evidence suggests that simpler tools—such as mHealth apps, SMS, and instant-messaging platforms—are frequently reported as highly feasible in LMIC hospital settings, often attributed to their lower cost, minimal infrastructure requirements, reliance on basic internet connectivity, and ease of integration into existing health care workflows. Reported facilitators include user-friendly interfaces, the convenience of remote monitoring, and the provision of continuous training, while reported barriers include poor network connectivity, high internet costs, inadequate digital literacy among patients, and limited integration with existing health system^7,25^. To be scalable, digital health innovations in LMICs may therefore need to be tailored to local contexts rather than imported wholesale from high-income countries^5,6^.

### The critical gap: absence of discharge readiness measurement

Despite the favourable outcomes reported across digital platforms, this review identified a major conceptual gap: none of the 11 included studies measured comprehensive discharge readiness as a primary outcome. Instead, the literature focused on individual components of readiness—disease knowledge, self-efficacy, or self-care behaviour—assessed independently. Discharge readiness is an important predictor of post-discharge outcomes, and inadequate readiness increases the risk of early unplanned readmissions, poor self-management, and worse clinical outcomes. Assessing readiness with validated tools such as the Readiness for Hospital Discharge Scale (RHDS) is therefore central to preparing patients and families for a safe transition from hospital to home^3,26^. Future trials should consider integrating digital education interventions with validated discharge-readiness assessment to better understand their impact on transitional care.

### Geographic disparities

Although the inclusion of studies from Brazil, Colombia, and Tunisia broadens the geographic representation, the evidence base remains concentrated in a small number of upper-middle- and lower-middle-income countries, predominantly China and Iran. Regions such as Southeast Asia (beyond Indonesia), South Asia, and sub-Saharan Africa remain markedly underrepresented. Because cultural context, technological infrastructure, and health literacy vary substantially across LMIC regions, the generalizability of findings from China and Iran to other settings should be interpreted with caution.

### Nursing and clinical implications

The findings of this review have direct implications for cardiovascular nursing practice. Nurses, who are in frequent contact with patients, play a pivotal role in delivering and managing digital education interventions^8,10^. By using platforms such as mobile apps and social media, they can provide continuous education that is independent of time and place, extending care beyond the hospital^10,16^. Integrating evidence-based digital education into post-discharge protocols allows providers to offer personalized goal-setting, remote monitoring, and timely psychosocial support—approaches identified in the literature as key strategies for supporting patient readiness and reducing preventable readmissions^4,9,10,26^.

## Conclusion

This scoping review mapped 11 studies evaluating digital education interventions designed to improve readiness among patients with coronary heart disease (CHD) in low- and middle-income countries (LMICs). The findings indicate that digital education interventions—primarily based on mobile applications, social media platforms such as WeChat and WhatsApp, SMS, web-based platforms, and digital multimedia including augmented reality—offer feasible modalities for improving individual components of patient readiness, including self-efficacy, quality of life, disease knowledge, and self-care behaviour.

These gaps notwithstanding, the evidence base has clear limitations. Geographically, it is concentrated in China and Iran, with limited representation from other LMIC regions such as Southeast Asia, sub-Saharan Africa, and South Asia. More importantly, no included study measured comprehensive discharge readiness as a primary outcome using a validated instrument.

Future research should prioritize the development of robust, culturally tailored digital education interventions across diverse LMIC settings to improve generalizability, and future controlled trials should pair these interventions with discharge-readiness assessment using validated instruments. Doing so would help close existing evidence gaps, support patients in the transition from hospital to home, and provide a firmer foundation for digital health policies responsive to the needs of CHD patients in LMICs.

## Acknowledgements

The authors gratefully acknowledge the support of the Faculty of Nursing, Hasanuddin University, in facilitating this research.

## Funding information

This research received no specific grant from any funding agency in the public, commercial, or not-for-profit sectors.

## Conflicts of interest

The authors declare no conflicts of interest.
